# A Bulk Retrospective Study of Robot-Assisted Stereotactic Biopsies of Intracranial Lesions Guided by Videometric Tracker

**DOI:** 10.3389/fneur.2021.682733

**Published:** 2021-08-04

**Authors:** Huan-Guang Liu, Yu-Ye Liu, Hua Zhang, Fan-Gang Meng, Kai Zhang, Guan-Yu Zhu, Ying-Chuan Chen, De-Feng Liu, Jian-Guo Zhang, An-Chao Yang

**Affiliations:** ^1^Department of Neurosurgery, Beijing Tiantan Hospital, Capital Medical University, Beijing, China; ^2^Department of Functional Neurosurgery, Beijing Neurosurgical Institute, Capital Medical University, Beijing, China; ^3^Key Laboratory of Computational Neuroscience and Brain-Inspired Intelligence (Fudan University), Ministry of Education, Beijing, China

**Keywords:** robotic, stereotactic surgery, biopsy, frameless, remebot robot

## Abstract

**Background:** Biopsies play an important role in the diagnosis of intracranial lesions, and robot-assisted procedures are increasingly common in neurosurgery centers. This research investigates the diagnoses, complications, and technology yield of 700 robotic frameless intracranial stereotactic biopsies conducted with the Remebot system.

**Method:** This research considered 700 robotic biopsies performed between 2016 and 2020 by surgeons from the Department of Functional Neurosurgery in Beijing's Tiantan Hospital. The data collected included histological diagnoses, postoperative complications, operation times, and the accuracy of robotic manipulation.

**Results:** Among the 700 surgeries, the positive rate of the biopsies was 98.2%. The most common histological diagnoses were gliomas, which accounted for 62.7% of cases (439/700), followed by lymphoma and germinoma, which accounted for 18.7% (131/700) and 7.6% (53/700). Bleeding was found in 14 patients (2%) by post-operation computed tomography scans. A total of 29 (4.14%) patients had clinical impairments after the operation, and 9 (1.29%) experienced epilepsy during the operation. The post-biopsy mortality rate was 0.43%. Operation time—from marking the cranial point to suturing the skin—was 16.78 ± 3.31 min (range 12–26 min). The target error was 1.13 ± 0.30 mm, and the entry point error was 0.99 ± 0.24 mm.

**Conclusion:** A robot-assisted frameless intracranial stereotactic biopsy guided by a videometric tracker is an efficient, safe, and accurate method for biopsies.

## Introduction

Clinically, when a patient develops an intracranial lesion that is not suitable for excision therapy and requires a tissue diagnosis to determine a proper treatment plan, the neurosurgery center prioritizes stereotactic biopsies. Stereotactic biopsy surgery can be used to treat multiple intracranial lesions, high-risk craniotomy areas—such as the brainstem and diencephalon (1, 2)—and patients whose poor health has led to intolerance to surgery. A histological diagnosis of a tumor is critical to adjuvant therapy, allowing follow-up treatment plans including radiotherapy, chemotherapy, and targeted drug therapy. Furthermore, a biopsy can distinguish between tumors, radiation necrosis, inflammation, and other lesion types.

Compared with frame-based biopsies, robot-assisted frameless stereotactic biopsies are highly efficient, safe, and simple and do not obstruct the frame (2–5), something currently recommended in many centers (6–11).

To provide further insight, this research investigates the diagnoses, complications, and technology yield of robotic biopsy operations for brain lesions. Specifically, it considers surgeries performed between 2016 and 2020 using the Remebot system guided by a videometric tracker.

## Materials and Methods

### Patients

This descriptive study included all patients who underwent a Remebot robot-assisted stereotactic biopsy (Beijing Baihui Weikang Technology Co., Ltd; Beijing, China) at Tiantan Hospital, China, between August 2016 and September 2020. Generally, clinical experts advise patients with intracranial lesions requiring clear categorization to undergo a biopsy to provide a basis for diagnosis and further treatment. However, a biopsy is not recommended for patients with severe coagulation dysfunction, diffuse lesions in the lower brainstem and oblongata, lesions rich in blood vessels or vascular lesions, or suspected acute bacterial inflammation (which can be spread through surgery).

All patients had preoperative and postoperative imaging examinations. This study was approved by the local ethics committee, and all patients or their relatives signed informed consent documents.

### Surgical Procedure

All operations were performed in the same center, the Department of Functional Neurosurgery at Tiantan Hospital, Beijing, China. One to two days before surgery, all patients underwent imaging information collection. To guarantee the visualization of the anatomical structures of interest, 3.0 Tesla magnetic resonance imaging (MRI, Siemens, Germany) for sagittal and axial volumetric T1-weighted (T1W1, slice thickness 1.0 mm, TR 6.4 ms, TE 3.0 ms, interslice gap 0 mm, flip angle 8°) and axial volumetric computed tomography (CT; 0.625 mm slice thickness) were performed in every patient. According to the intracranial lesion, the doctors would choose axial and coronal volumetric T2-weighted MRI (T2W1, 2.0 mm slice thickness), sagittal and axial volumetric T1-weighted fluid attenuated inversion recovery (FLAIR, 1.0 mm slice thickness), and other metabolic imaging or vascular imaging sequence. A full head scan with enhanced CT was applied for patients with metal implants (steel plates, dentures, etc.) instead of MRI, with a thickness of 0.625 mm.

**Figure 1:**
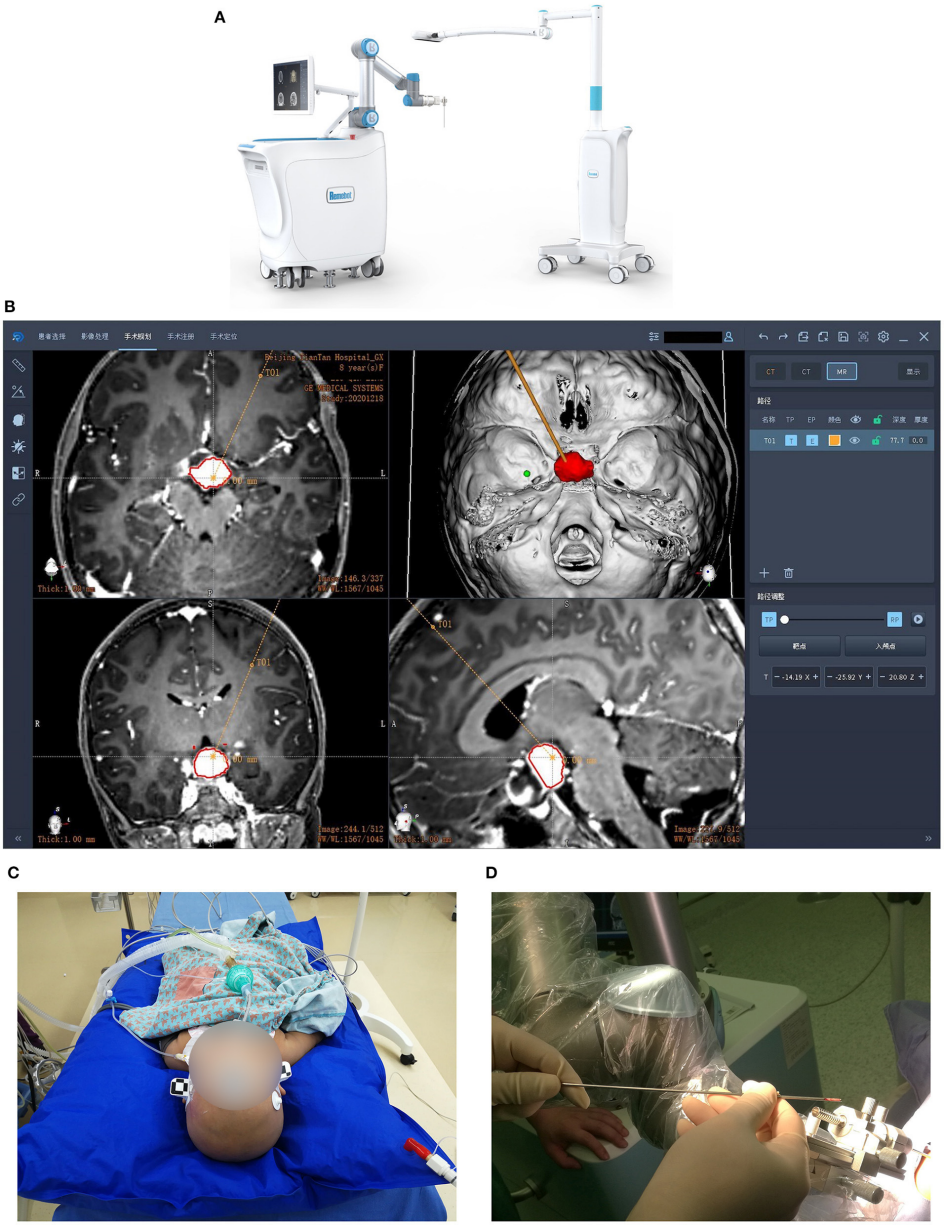
Images of the Remebot and surgical workflow. **(A)** The Remebot device comprises a videometric tracker, a planning station, and a robotic arm. **(B)** The trajectories were planned according to the fusion of preoperative MRI and CT images. **(C)** The videometric tracker scanned the optical marker pasted on the patient's head to complete registration. Local anesthesia for most patients while general anesthesia for children and some uncooperative patients. **(D)** Biopsies were taken at multiple points.

The Remebot robotic device comprises a videometric tracker, a planning station, and a robotic arm (Figure 1A). The videometric tracker (MicronTracker, ClaroNav, Canada), featuring three stereotactic cameras positioned by an independent stand, was installed above the patient's head, enabling the optical markers to be detected within the tracker's field of measurement.

Next, the image data was imported into the planning station to construct three-dimensional models, determine the surgical target, and plan the trajectories (Figure 1B). On the day of surgery, the patient was fixed by a head holder and most of them received local anesthesia, while children and uncooperative adults received general anesthesia.

The videometric tracker then automatically scanned the markers pasted on the patient's head to complete tracker-to-image registration. Following the operation's registration, the robotic arm moved to determine the skull entry point (Figure 1C). After the operation site and the robotic arm were disinfected, the surgical drill needle was installed. Biopsies were taken in four directions at 3, 6, 9, and 12 o'clock when the biopsy needle slowly approached the target position (Figure 1D). According to the actual situation, additional tissues were taken at 4, 8, and 12 o'clock, 5 mm above or below the target point. These biopsies were sent to pathology. Finally, all patients were reexamined by CT or MRI.

### Data Collection

We collected basic information about patients, including name, gender, age, etc. We counted the number of histological diagnoses after biopsy, and calculated the positive rate of biopsy. For histological identification, we used the 2016 World Health Organization classifications, which include data based on molecular biology. Complications represent the safety of surgery, we count the types, treatment plan and prognosis of complications after surgery. The operation time represents the efficiency of the operation, including registration time and operation time. The error of the cranial point and the error of the target point represent the accuracy of the operation. Descriptive analysis is presented as number and percentages for enumeration data, and the measurement data is expressed by the mean ± standard deviation.

## Results

### Patients

A total of 700 patients who underwent robot-assisted stereotactic biopsies were treated in Tiantan Hospital from August 2016 to September 2020. This included 357 males and 343 females aged 16 to 72 years (average age of 47.6 ± 16.5 years).

### Histological Diagnoses

The positive rate for biopsies was 98.2% (687/700). Of the 700 patients who underwent biopsies, 439 were histologically diagnosed with gliomas, accounting for 62.7% of the patients. The most common gliomas were glioblastomas, followed by anaplastic oligodendrocytomas, ligobranched astrocytomas, and diffuse midline gliomas. There were 194 cases of other tumor types (27.7%), with lymphoma and germinoma being most common, accounting for 18.7% (131/700) and 7.6% (53/700) of cases. Additionally, there were 47 cases of focal inflammation, six cases of radiation necrosis, and one case of nerve demyelination, all of which were critical for differential diagnosis. However, 13 patients could not receive a clear histological diagnosis, potentially because the biopsy tissue was located at the edge of the lesion or the patient moved their head while under local anesthesia, producing non-specific histological diagnoses that could not be confirmed. Table 1 provides details of the results discussed in this paragraph.

**Table 1:** Breakdown of observations and diagnoses.

Glioma			
Glioblastoma	130	Anaplastic oligodendrocytoma	100
Oligobranched astrocytoma	90	Diffuse midline glioma	45
Ungraded high-grade glioma	24	Ungraded low-grade glioma	22
Oligodendroglioma	13	Ganglioglioma	9
Glial cell proliferation	6	Total	439 (62.7%)
Other Tumors			
Lymphoma	131	Germinoma	53
Metastasis	3	Meningioma	2
Melanoma	2	Craniopharyngioma	2
Schwannoma	1	Total	194 (27.7%)
Others			
Inflammation	47	Radiation necrosis	6
Demyelination	1	Total	54 (7.7%)
Missing	13	1.8%	

### Complications

The main complications were bleeding, clinical impairment, and epilepsy (Table 2). There were no infectious complications.

**Table 2:** Data for postoperative complications.

Bleeding		(14 patients, 2%)
Bleeding location	Intracerebral hemorrhage	12
	Epidural bleeding	2
Treatment	Hematoma evacuation	7
	Conservative treatment	7
Prognosis	Rehabilitation	11
	Hemiplegia	1
	Death	2
Clinical impairment		(29 patients, 4.14%)
Prognosis	Rehabilitation	28
	Death	1
Epilepsy	Focal epilepsy	(9 patients, 1.29%)

Postoperative CT scans revealed bleeding in 14 patients (2%), including 12 cases of intracerebral hematoma and two cases of epidural hematoma. All bleeding resulted from supratentorial hemorrhaging. If blood volume was ≥30 ml or bleeding had not stopped, a second operation was performed to remove the hematoma. Of the seven patients who underwent the second surgery, six made complete recoveries, including two patients with epidural hematoma and four with intracerebral hematoma. One intracerebral hematoma patient developed hemiplegia after the hematoma evacuation (1/700, 0.14%). Seven patients were treated conservatively, with five entering complete remission and two died unfortunately.

Twenty-nine patients developed aggravated neurological symptoms but generally recovered through conservative treatment. One patientdied 3 months later, having shown clinical impairment following surgery.

Nine people experienced intraoperative epilepsy with local anesthesia, prompting immediate treatment with midazolam. If the biopsy site was located in the functional area, patients were regularly treated with levetiracetam for 3 months post-operation, with those experiencing epilepsy during the operation requiring long-term medication. No postoperative epilepsy was observed.

A total of three patients died following their biopsies—two from bleeding, and one due to worsening neurological symptoms—indicating a post-biopsy mortality rate of 0.43%. The two patients who died of postoperative bleeding were both older than 75 years old and were diagnosed with glioblastoma. Due to advanced age, poor physical condition, and large tumor volume, family members agreed with experts' recommendations for conservative treatment after bleeding, and both patients unfortunately died. One patient with a midbrain lesion slipped into a coma immediately after the operation, with a pathological diagnosis of glioblastoma. Experts recommend palliative treatment because of the high risk and little benefit of surgery, and eventually died 3 months later.

### Technical and Workflow Aspects

Technical data for the robotic biopsies are shown in Table 3. The registration time was within 2 min guided by the videometric tracker. Operation time—from marking the cranial point to suturing the skin—was 16.78 ± 3.31 min, indicating a range of 12 to 26 min. The target error was obtained by comparing the target points in the trajectories with the actual target position, which was 1.13 ± 0.30 mm (ranging from 0.57 to 1.78 mm). The entry point error was 0.99 ± 0.24 mm (ranging from 0.56 to 1.73 mm), indicating a minimal difference between the planned and actual skull entry points.

**Table 3:** Data for robotic biopsy surgeries.

Operation time (min)		16.78 ± 3.31
Error	Entry point error (mm)	0.99 ± 0.24 (0.56–1.73)
	Target error (mm)	1.13 ± 0.30 (0.57–1.78)

## Discussion

The study of surgical robotics has developed rapidly in recent years, especially in the field of functional neurosurgery, with applications including deep brain stimulation surgery, stereo-electroencephalography, cerebrospinal fluid shunts, ventriculoscopic surgery, and stereotactic biopsies. For example, Zanello et al. assessed the conditions of 377 patients with various types of gliomas following robot-assisted serial stereotactic biopsies (12), Hamzah et al. reported a series of 102 frameless stereotactic biopsies using a robotic device (6), and Lefranc et al. reported 100 cases of surgery using the ROSA robotic device (7). As one of the largest neurosurgery centers in China, our hospital receives a large number of intracranial lesion patients, many of whom require biopsies. Accordingly, this study investigated the diagnoses, complications, and technical yield of 700 instances of robotic biopsy surgery for brain lesions performed between 2016 and 2020. As far as we know, this is the largest single-center study of robot-assisted stereotactic biopsy.

Following previous reports of bulk biopsy records observing positive rates between 91.3 and 100% for biopsies (6, 7, 11, 13, 14), the positive rate among our 700 patients was 98.2%. Notably, Hamzah et al. reported a positive rate of 92.2% in their study of 102 biopsies conducted using the Neuromate robot, with glial and glioneuronal tumors constituting the most common histological diagnoses (6), although other pathological findings were similar to ours. Meanwhile, Lefranc et al. reported 97 cases (97%) establishing histological diagnoses (7).

Regarding complications, compared to our bleeding rate of 2%, the literature has recorded bleeding rates between 0 and 13.1% (1, 3, 6, 11, 15–17). One report considering 379 patients observed 12 cases (3.2%) presenting intracerebral hematoma of ≥20 ml, three of whom needed surgical cleaning (12, 15), with researchers suggesting that the biopsy trajectory of most patients with hemorrhaging resulted from contact with blood vessels or cerebral sulcus (15). Although the safety of biopsies has generally been proven high, that brainstem and diencephalon bleeding rates can reach 13.1% may be due to the surgery's unavoidable movement through complex and abundant blood vessel tissues (1). For example, Malone et al. analyzed 7,514 intracranial biopsies conducted across multiple centers, finding intracranial hemorrhaging to be the most common complication (5.8%), with multivariate logistic regression analyses associating hemorrhaging with advanced age (≥60 years) and hydrocephalus (17). In our research, most bleeding patients were diagnosed with glioblastoma, and patients with brainstem biopsy had no bleeding complications. We believe that slow injection, small tissue quantities, controlling intraoperative blood pressure, and using small-diameter needles for dangerous tumors and bleeding-prone sites would helpfully reduce the bleeding rate. Meanwhile, if bleeding does occur, the hemostatic effect of a gelatin sponge is better than that of hemocoagulase.

Our investigation of the surgical experience revealed that deep, high-grade gliomas are prone to bleeding, and paraventricular tumors easily break into the ventricles. Therefore, the needle should be injected slowly, as little tissue as possible should be taken, and blood pressure should be controlled during the operation. If bleeding is found, the biopsy operation should be stopped, and locally applying a gelatin sponge would be more beneficial than using hemocoagulase. Tumors with high-risk coefficients should be operated on with fine needles. High-grade gliomas and lymphomas are prone to irritation, aggravating edema, and worsening clinical symptoms. If progress occurs, mannitol plus hormone treatment should be used as soon as possible according to the freezing results. If lymphoma is suspected, postoperative edema can be treated with 500 mg methylprednisolone or rituximab. Low-grade gliomas near the cortex or the functional area often promote intraoperative seizures. To avoid this situation, we advocate a gentle and shortened operation minimizing electric knife use. For patients with a tumor occupying space near the cortex, it is necessary to prepare the intravenous pathway in advance and communicate with the anesthesiologist to prepare the sedative midazolam.

It is also worth considering our findings in the context of breakthroughs robot-assisted stereotactic biopsies have made for accuracy and usability. Few studies provided accuracy measures for the entry and target point separately. Elsewhere, application of the iSYS-1 robot produced an entry point error of 2 mm (ranging from 0.2 to 3.8 mm) and a target error of 1.06 mm (ranging from 0.1 to 4 mm) in 39 patients (9), and 1.3 mm (ranging from 0.2 to 2.6 mm) at entry and 0.9 mm (ranging from 0.0 to 3.1 mm) at the target point in 25 patients from another research (10). Dlaka et al. reporting 32 biopsy surgeries by the RONNA G3 robot calculated an entry point error of 1.42 ± 0.74 mm and target point error of 1.95 ± 1.11 mm (18). In addition, Alice Goia et al. reported 44 lead implantations using the ROSA robot for deep brain stimulation surgery, observing target errors of 0.81 ± 0.51 mm on the right and 1.12 ± 0.75 mm on the left (19). This indicates that the accuracy of this study's Remebot-assisted biopsy procedure is comparable to that of other available robots, with a mean entry point error was 0.99 and a mean target error as low as 1.13 mm.

The Remebot system includes a six-joint robotic arm and an independent videometric tracker—in this case, the MicronTracker—which uses visible lighting to detect and track objects of interest marked by a target pattern that comprises high-contrast black-and-white interlaced regions called Xpoints (Figure 1C). The tracker's calibration accuracy is 0.2 mm, identical to that of other optical tracking systems. Notably, the MicronTracker has been used in rigid tissue surgical navigation in many disciplines (20–22). The Remebot system provides an automatic and rapid workflow to facilitate registration procedures and eliminate human error. Compared with the artificial bone-loaded benchmark, the new frameless optical registration is more convenient (14).

The most obvious limitation of our study is the lack of follow-up treatment data. Since biopsy, radiotherapy, chemotherapy and tumor resection belong to different departments, relevant data are unfortunately not available. It would be interesting to explore the subsequent treatment of patients with biopsies.

## Conclusion

This study reported on the 700 robot-based frameless biopsies conducted in our center between 2016 and 2020. The positive rate of biopsies was high, and the incidence of complications was acceptable, confirming that robot-assisted frameless stereotactic biopsies are an accurate, efficient, and safe biopsy method.

## Data Availability Statement

The original contributions presented in the study are included in the article/supplementary material, further inquiries can be directed to the corresponding author/s.

## Ethics Statement

The studies involving human participants were reviewed and approved by Medical Ethics Committee of Tiantan Hospital. Written informed consent for participation was not required for this study in accordance with the national legislation and the institutional requirements.

## Author Contributions

J-GZ and A-CY contributed to conception and design of the study. H-GL and HZ organized the database. Y-YL performed the statistical analysis and wrote the first draft of the manuscript. F-GM, KZ, G-YZ, Y-CC, and D-FL wrote sections of the manuscript. All authors contributed to manuscript revision, read, and approved the submitted version.

## Conflict of Interest

The authors declare that the research was conducted in the absence of any commercial or financial relationships that could be construed as a potential conflict of interest.

## Publisher's Note

All claims expressed in this article are solely those of the authors and do not necessarily represent those of their affiliated organizations, or those of the publisher, the editors and the reviewers. Any product that may be evaluated in this article, or claim that may be made by its manufacturer, is not guaranteed or endorsed by the publisher.
